# BioMedKG: multimodal contrastive representation learning in augmented BioMedical knowledge graphs

**DOI:** 10.3389/fsysb.2025.1651930

**Published:** 2025-12-08

**Authors:** Tien Dang, Viet Thanh Duy Nguyen, Minh Tuan Le, Truong-Son Hy

**Affiliations:** 1 Department of Computer Science, The University of Alabama at Birmingham, Birmingham, AL, United States; 2 Department of Computer Science, Washington University in St. Louis, St. Louis, MO, United States

**Keywords:** biomedical knowledge graphs, multimodal, graph representation learning, graph contrastive learning, medical languagemodels, data augmentation, link prediction, drug repurposing

## Abstract

Biomedical Knowledge Graphs (BKGs) integrate diverse datasets to elucidate complex relationships within the biomedical field. Effective link prediction on these graphs can uncover valuable connections, such as potential new drug-disease relations. We introduce a novel multimodal approach that unifies embeddings from specialized Language Models (LMs) with Graph Contrastive Learning (GCL) to enhance intra-entity relationships while employing a Knowledge Graph Embedding (KGE) model to capture inter-entity relationships for effective link prediction. To address limitations in existing BKGs, we present PrimeKG++, an enriched knowledge graph incorporating multimodal data, including biological sequences and textual descriptions for each entity type. By combining semantic and relational information in a unified representation, our approach demonstrates strong generalizability, enabling accurate link predictions even for unseen nodes. Experimental results in PrimeKG++ and the DrugBank drug-target interaction dataset demonstrate the effectiveness and robustness of our method in diverse biomedical datasets. Our source code, pre-trained models, and data are publicly available at https://github.com/HySonLab/BioMedKG.

## 1 Introduction

**FIGURE 1:**
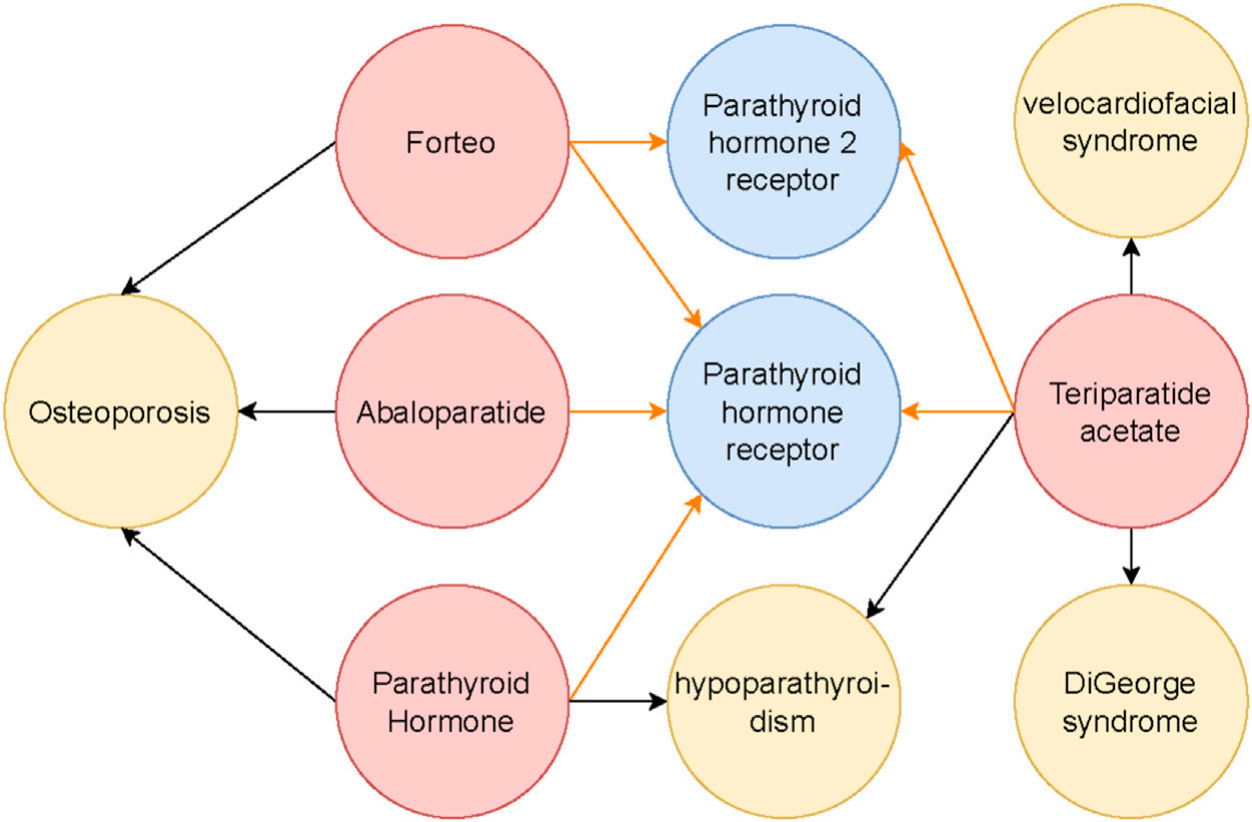
The subgraph illustrates the interactions surrounding the Parathyroid hormone receptor and its connections to related drugs and diseases. Different entity types are color-coded: red nodes represent drugs, blue nodes indicate genes or proteins, and yellow nodes denote diseases. Black arrows depict drug-treatment relationships with diseases, while orange arrows represent drug-receptor interactions. This subgraph is a focused segment of a broader Biomedical Knowledge Graph, which captures the complex interconnections among various biological entities.

BKGs are structured networks that represent intricate relationships among biological entities such as genes, proteins, diseases, and drugs (see Figure 1). Accurate link prediction within these graphs is crucial for identifying hidden relationships, discovering potential therapeutic targets, and suggesting drug repositioning opportunities (Nicholson and Greene, 2020; Zitnik et al., 2018; Ngo et al., 2022). These capabilities can significantly accelerate biomedical research, leading to faster clinical advancements and more effective treatments.

Despite their potential, generating consistent and effective node representations for link prediction in BKGs remains a challenging task. A promising strategy to address this issue is to improve the existing knowledge base by integrating rich, multimodal domain-specific data associated with these entities.

Recent advances show that pre-trained LMs can act as foundational knowledge bases, storing vast amounts of factual information (Petroni et al., 2019; He et al., 2024; Zhao et al., 2024; Jiang et al., 2024; Chen, 2023). When used as initial embeddings, LMs provide a strong foundation for downstream tasks by incorporating pre-existing knowledge from biomedical texts and databases (Wang et al., 2023). These models offer rich semantic information that can enhance the learning of graph representations. However, previous work on BKGs (Daza et al., 2023; Lam et al., 2023) has focused mainly on using single-modality node representations for each node type (e.g., amino acid sequences for proteins, SMILES strings for drugs and textual descriptions for diseases), overlooking the potential to integrate multiple modalities for each node type. Moreover, while LM-derived embeddings serve as initial representations for knowledge graphs, they often lack graph topology, necessitating fine-tuning to effectively capture graph structure.

In this work, we propose a novel pre-trained node representation model designed to enhance link prediction performance in BKGs. Our comprehensive framework leverages the capabilities of LMs to generate robust entity representations while seamlessly integrating multimodal information to enrich the contextual understanding of relationships within the graph. Specifically, we unify LM-derived embeddings for each entity and employ GCL to optimize intra-node relationships by enhancing mutual information within individual node types. Additionally, we utilize a KGE model to capture inter-node information between different biological entities. A key feature of our approach is its generalizability, as the node embeddings generated by our framework encapsulate both semantic information from LMs and relational information from GCL. This dual integration ensures that the embeddings maintain a rich contextual understanding, allowing the framework to generate meaningful representations even for unseen nodes, thereby facilitating more accurate link prediction for novel entities.

However, our approach requires a BKG with well-defined node attributes, which are absent in most existing BKGs that lack comprehensive attributes for each entity type (Chandak et al., 2023; Walsh et al., 2020). To address this limitation, we introduce *PrimeKG++*, an enriched knowledge graph that builds on PrimeKG (Chandak et al., 2023). PrimeKG++ enhances the original dataset by incorporating biological sequences for each entity type: amino acid sequences for proteins, nucleic acid sequences for genes, and SMILES strings for small molecules, along with comprehensive textual descriptions. This integration diversifies node attributes and improves the overall utility of the knowledge graph, providing a valuable public resource for future research in biomedical knowledge graphs.

It is important to clarify that the primary focus of this paper is not on achieving state-of-the-art results in downstream tasks such as link prediction. Instead, we aim to propose a pre-trained node representation model and demonstrate its effectiveness through comprehensive experiments. To evaluate this, we used existing models with and without our pre-trained node representations as initial inputs. Our experiments show that our pre-trained node representations lead to significant performance improvements compared to random initialization or Direct LM-derived embeddings. By leveraging SOTA models for link prediction, we ensured that our comparisons were rigorous and meaningful, demonstrating the added value of our pre-trained node representations within an established and high-performing framework.

The contributions of this work are summarized as follows.

- We propose a comprehensive framework that leverages LMs and GCL to create robust, multimodal node embeddings for BKGs.
- We present PrimeKG++, an augmented biomedical knowledge graph enriched with biological sequences and textual descriptions, which provides a comprehensive resource for our work and the biomedical research community.
- We validate the effectiveness and generalizability of our approach through extensive empirical results.

## 2 Related works

### 2.1 Biomedical knowledge graphs

Biomedical Knowledge Graphs (BKGs) integrate diverse biological and clinical data to model complex relationships among entities such as genes, proteins, drugs, and diseases. Several large-scale BKGs have been developed to facilitate biomedical discovery and reasoning. Hetionet (Himmelstein et al., 2017) is an early integrative graph that connects biomedical entities from 29 databases, effectively enabling drug repurposing and disease association studies. However, it primarily focuses on relational structure and contains limited multimodal node attributes. BioKG (Walsh et al., 2020) extends this idea by incorporating additional biomedical entities (e.g., pathways, side effects) and unifying them under a consistent schema, but it still relies mainly on symbolic relations and lacks comprehensive textual or sequence-based metadata. PrimeKG (Chandak et al., 2023) advances the field by introducing a multimodal precision medicine graph that links molecular, clinical, and textual information, yet its node attributes remain incomplete–particularly for genes and proteins, which lack sequence or functional annotations. To address these gaps, we constructed PrimeKG++, an enhanced version of PrimeKG that integrates biological sequences (e.g., amino acid, nucleotide, and SMILES representations) and textual descriptions for key entity types, including drugs, genes, and proteins. Compared to prior BKGs, PrimeKG++ provides richer multimodal context and more fine-grained entity attributes, supporting improved representation learning and interpretability in biomedical applications.

### 2.2 Knowledge graph embedding

In the field of BKGs, link prediction research aims to uncover connections among biological entities by analyzing their existing links and attributes (Menon and Elkan, 2011; Zitnik et al., 2018; Hansel et al., 2023; Wang et al., 2021; Fu et al., 2021). Knowledge graph embeddings, representing entities and relations as vectors, have gained popularity for this task. Although traditional models, such as ComplEx (Trouillon et al., 2016) and RotatE (Sun et al., 2019) have shown promising results in this link prediction task, two key constraints hinder them: first, they focus solely on the graph structure, ignoring valuable entity attribute information; and second, their reliance on predetermined embeddings for mapping entities and relations in the lookup table complicates integration with new entities. These constraints motivate us to construct a heterogeneous biomedical knowledge graph with multimodal metadata.

### 2.3 Biomedical language model

In BKGs, entities can possess different modalities, such as text or biological sequences. Essentially, a molecular sequence is the exact order of smaller units (monomers) that make up a large molecule (biopolymer). Similarly to a textual description, it inherently possesses a sequential relationship that LMs can effectively process. Recent methods rely on pre-trained language models such as BERT (Devlin et al., 2019) as the backbone for the attribute encoder. Protein sequences, which are strings of amino acid letters, can be processed effectively by models such as ESM-2 (Lin et al., 2023) and ProteinBERT (Brandes et al., 2022). For genes, which are represented by nucleotide sequences, specific language models such as Nucleotide Transformers (Dalla-Torre et al., 2023) and DNABERT (Ji et al., 2021) are required. Chemical structures are often represented using SMILES strings, a linear text format, which can be interpreted by models such as BARTSmiles (Chilingaryan et al., 2024) and MoLFormer (Ross et al., 2022). For textual descriptions in the biomedical domain, models such as BioGPT (Lewis et al., 2020) and BioBERT (Lee et al., 2020) are used to extract high semantic meaning, providing improved understanding and analysis of biomedical text. These findings inspire us to explore the potential of LMs to extract semantic information from node features in BKGs.

### 2.4 Graph contrastive learning

Many Graph Neural Networks rely on supervised learning with labeled data, which is costly and labor-intensive. To address this, some studies (e.g., DGI (Veličković et al., 2018), MVGRL (Hassani and Khasahmadi, 2020), GMI (Peng et al., 2020), and GRACE (Zhu et al., 2020) use contrastive learning techniques, introducing Graph Contrastive Learning for self-supervised graph representation learning. These methods aim to maximize mutual information between an anchor node and its semantically similar counterparts while minimizing it for dissimilar ones. In recent years, contrastive learning has gained traction in knowledge graph embedding. KGCL (Yang et al., 2022) integrates knowledge graph learning with user-item interaction modeling through a joint self-supervised learning approach, improving robustness and addressing data noise and sparsity in recommendation systems. KE-GCL (Zhang and Li, 2022) incorporates contextual descriptions of entities and proposes adaptive sampling to refine the contrastive learning of the knowledge graph. MCLEA (Lin et al., 2022) unifies information from various modalities and uses contrastive learning for discriminative entity representations. However, multimodal contrastive learning has not yet been explored in BKGs. In this paper, we present a novel graph representation learning framework that incorporates contrastive learning for biomedical knowledge graphs.

## 3 PrimeKG++: an augmented knowledge graph

PrimeKG (Chandak et al., 2023) is a multimodal knowledge graph tailored for precision medicine, comprising more than 100,000 nodes across various biological scales. It features more than four million relationships between these nodes, categorized into 29 distinct edge types. We selected PrimeKG for its enriched disease nodes, which are annotated with clinical descriptors sourced from trusted medical authorities. This enrichment provides a strong foundation for applying LM-derived embeddings, enabling more precise and contextually relevant analyses in biomedical research. However, PrimeKG exhibits limitations, particularly in its lack of detailed contextual or descriptive information for other biological entities such as genes and proteins. This limitation reduces the graph’s ability to fully capture the intricate interactions and functions inherent in biological systems.

To address these limitations, we developed PrimeKG++, an enhanced version of PrimeKG that integrates detailed multimodal information for three key node types: gene/protein, drug, and disease. PrimeKG++ categorizes drug data into two subtypes: molecules, represented with SMILES strings, and antibodies, identified by amino acid sequences. For the gene/protein node type, it includes protein-coding genes, annotated with amino acid sequences, and non-coding genes, represented with nucleotide sequences. Descriptive textual information is collected for all subtypes of drugs and genes/proteins, providing richer biological and functional context. These enhancements are carefully linked to authoritative sources such as Entrez Gene (Maglott et al., 2010) for genes and proteins and DrugBank (Knox et al., 2024) for drugs, using consistent PrimeKG identifiers.

To illustrate these improvements, Table 1 presents representative examples from the Drug and Gene/Protein node types. Each example demonstrates how PrimeKG++ enriches the original PrimeKG by incorporating biological sequences and descriptive annotations from domain-specific databases. These additional multimodal attributes enable a more comprehensive and interpretable representation of molecular and genetic entities, thereby improving the graph’s capacity to model complex biological relationships.

**TABLE 1:** Example entities in PrimeKG++ illustrating additional multimodal attributes integrated for each major node type and subtype.

Node type	Subtype	Node ID	Biological sequence	Description
Drug	Small molecule	DrugBank: DB01001	(SMILES) CC(C)(C)NCC(O)C1 = CC(CO) = C(O)C=C1	Salbutamol (Albuterol [USAN]) is a short-acting, selective beta2-adrenergic receptor agonist used in the treatment of asthma and COPD.
	Antibody	DrugBank: DB12688	Heavy: MEVQLVESGG … Light: MDIQMTQTT …	Moxetumomab pasudotox, a monoclonal antibody linked to a toxin, identifiable by the “-mab” suffix in its name
Gene/Protein	Protein-coding gene	Entrez: 30,844	(Amino acid sequence) MFSWMGRQAG …	EHD4, enables cadherin binding activity and is involved in endocytic recycling
	Non-coding gene	Entrez: 100,126,333	(Nucleotide sequence) AACTGCCCTC …	MIR708, a microRNA, which is a short, non-coding RNA molecule involved in gene regulation

## 4 Methods

**FIGURE 2:**
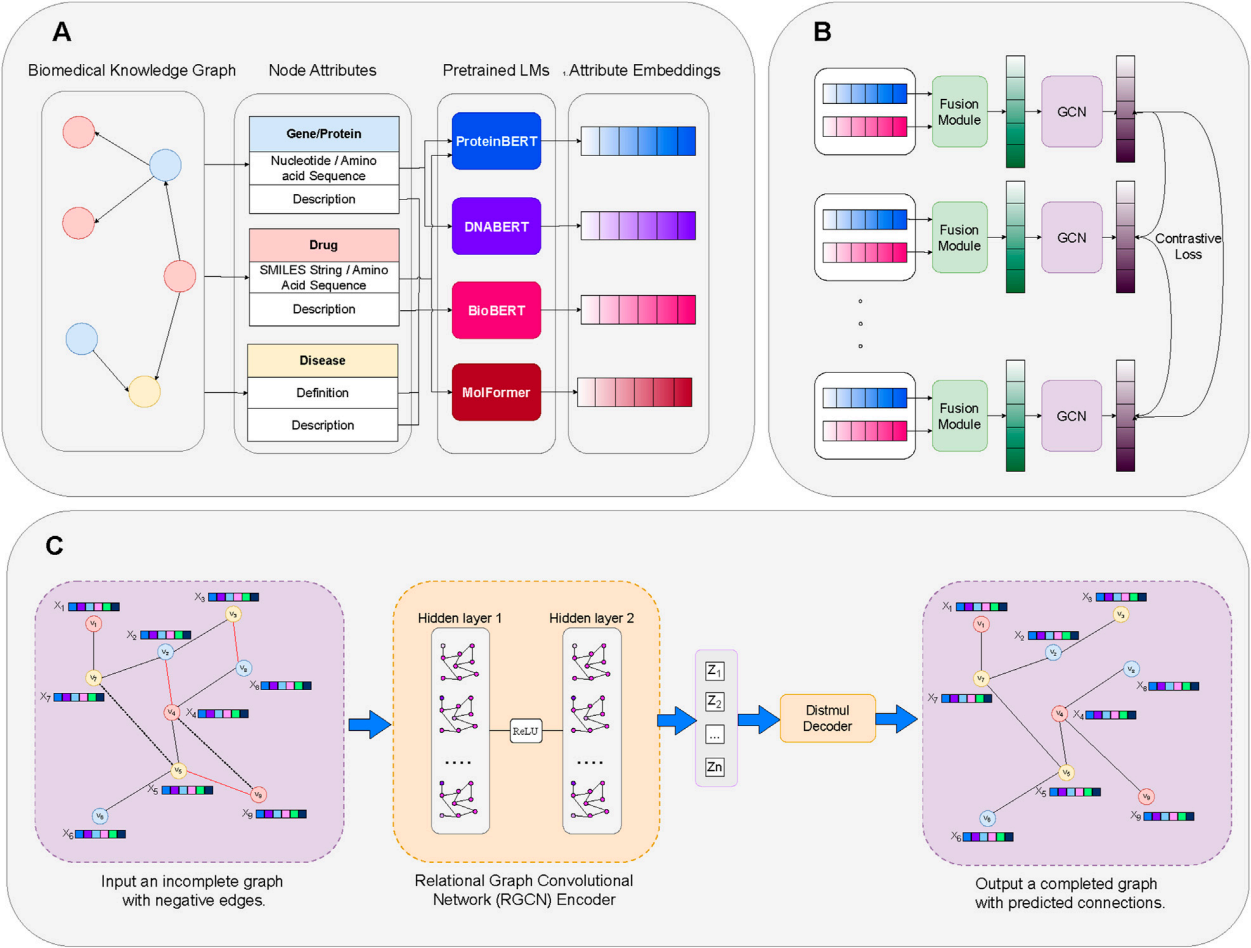
Overview of our proposed framework. **(A)** Modality Embedding: Creating node attribute embeddings through domain-specific LMs. **(B)** Contrastive Learning: Enhancement of LM-derived embeddings for specific node attributes of the same type through Fusion Module and Contrastive Learning. **(C)** Link Prediction on KG Embedding: Utilizing the enhanced embeddings to perform link prediction tasks through a Knowledge Graph Embedding (KGE) model that learns relationships and enhances semantic information across distinct node types.

Our framework is illustrated in Figure 2. Initially, we generate embeddings for each node type’s modalities using their corresponding Language Models (Section 4.2). The embeddings of these modalities are then integrated into a unified embedding space via the Fusion Module (Section 4.3). Subsequently, the Graph Contrastive Learning module enhances relationships within homogeneous biomedical subgraphs, facilitating intra-node learning (Section 4.4). Finally, the Knowledge Graph Embedding module refines these embeddings through link prediction tasks to enhance learning across different node types, fostering inter-node learning (Section 4.5).

### 4.1 Preliminaries

In the context of knowledge graphs where entities have associated attributes across various modalities, we define a Biomedical Knowledge Graph as G=(V,R,E,D,d) . Here, V denotes the set of nodes, collectively $\left\{v_{1},\ldots ,v_{n}\right\}$ , with n being the number of entities, R denotes the set of relations, and E consists of triples (h,r,t) where h,t∈V and r∈R . The elements h , r , and t represent the head, relation, and tail of a triple, respectively. D represents a dataset with attributes of entities, where each type of entity has specific attributes relevant to its biological role. The partial function $d:V_{d}\to D$ maps a subset of entities $V_{d}\subseteq V$ , which have available attribute data, to their respective attributes, with $d\left(v_{i}\right)$ retrieving the attribute data for an entity $v_{i}$ . This schema allows for customized attribute representation, accommodating the diverse and specific data needs of different entity types within the graph. The complete list of notations is provided in Table 2.

**TABLE 2:** List of notations.

Notation	Definition
G	Knowledge graph
V	Set of nodes: $\left\{v_{1},v_{2},\ldots ,v_{n}\right\}$
R	Set of relations: $\left\{r_{1},r_{2},\ldots ,r_{m}\right\}$
E	Set of triples (h,r,t) : $\left\{\left(h_{1},r_{1},t_{1}\right),\ldots ,\left(h_{k},r_{k},t_{k}\right)\right\}$
D	Dataset with attributes: $\left\{D_{1},D_{2},\ldots ,D_{p}\right\}$
$d:V_{d}\to D$	Function mapping entities to attributes: $d\left(v_{i}\right)\to D_{i}$
$e_{i}$	Modality-specific encoder: $e_{i}:D_{i}\to X_{i}$
$X_{i}$	Embedding space of modality i : $R^{d_{i}}$
$x_{i}$	Modality-specific embedding: $x_{i}\in R^{d_{i}}$
E	Encoder function: $E\left(x_{i}\right)\to R^{D}$
$h_{i}$	Unified embedding: $h_{i}\in R^{D}$
A	Adjacency matrix: $A\in R^{n\times n}$
X	Node feature matrix: $X\in R^{n\times d}$
Z	Latent node representation: $Z\in R^{n\times d}$
R	Set of relations: $R\in R^{m\times d}$
α	Weight for regularization term: 0.01
h	Head entity in a triple: h∈V
t	Tail entity in a triple: t∈V
r	Relation in a triple: r∈R

### 4.2 Modality encoding

We utilize a set of k modality-specific *encoders*, $\left\{e_{1},\ldots ,e_{k}\right\}$ , where each encoder $e_{i}$ corresponds to a pre-trained Language Model for a specific attribute modality $D_{i}\subseteq D$ . Each encoder $e_{i}$ maps its respective attribute data into a distinct embedding space $X_{i}$ , formally represented as $e_{i}:D_{i}\to X_{i}$ . Selection of these LMs involves ensuring uniformity in embedding sizes and balancing the computational complexity with the desired level of accuracy, to optimize both integration across modalities and overall system efficiency. Specifically, we use ProtBERT (Brandes et al., 2022) for protein sequences, DNABERT (Ji et al., 2021) for gene sequences, MolFormer (Ross et al., 2022) for SMILES strings of molecules and BioBERT (Lee et al., 2020) for descriptions of all types of entities. These choices ensure that the attribute embeddings leverage domain-specific knowledge encoded in the LMs, thereby enhancing the quality and applicability of the generated embeddings. During training, the LMs are frozen to reduce the number of trainable parameters. Another concern is that Knowledge Graphs are often incomplete due to undisclosed or overlooked facts. For these cases, we randomly initialize the attribute embeddings.

### 4.3 Modality fusing

On top of proposing a collection of features collectively representing each node type, we propose a Fusion Module designed to effectively integrate diverse modalities of node-specific features into a common embedding space. Formally, for an entity $v_{i}\in V$ with modality-specific embeddings $x_{1},x_{2},\ldots ,x_{M}$ , where each $x\in R^{d}$ . The encoder function E projects a concatenation of these embeddings in the space $R^{d\times M}$ into a common embedding space $R^{d}$ , producing a unified embedding $u_{i}$ as follows:

$$u_{i}=E\left(x_{1},x_{2},\ldots ,x_{M}\right),\;u_{i}\in R^{d},$$

where each $u_{i}\in R^{D}$ . This approach allows each modality to be represented in the same dimensional space, facilitating further analysis or fusion at a subsequent stage of the model.

To achieve effective integration of these modality-specific embeddings, we utilize Attention Fusion (Vaswani et al., 2017) and Relation-guided Dual Adaptive Fusion (ReDAF) (Zhang et al., 2024). These fusion methods determine the contribution of each modality before combining them into a unified representation, which is essential because different modalities may carry varying levels of importance depending on the context. By assigning appropriate weights to each modality, the model can better capture the most relevant information, resulting in a more accurate and meaningful representation of the entity. Regardless of the fusion method used, a simple mean operation is applied at the final stage to ensure a balanced integration of the multi-modal embeddings, allowing for a cohesive representation of each entity. The detailed mechanisms and formulations of these techniques are provided in Section 4.3.1 and Section 4.3.2.

#### 4.3.1 Attention fusion

The Attention Fusion layer integrates diverse modality-specific embeddings into a unified representation by employing attention mechanisms. This approach enables the model to dynamically weigh the importance of each modality based on its relevance to the task, thus enhancing the overall quality of the integrated embeddings.

Formally, consider an entity v∈V with modality-specific embeddings $x_{1},x_{2},\ldots ,x_{M}$ , where each $x_{i}\in R^{d_{i}}$ . The Attention Fusion layer projects these embeddings into a common space $R^{D}$ , and then uses attention scores to combine them.

First, each modality-specific embedding $x_{i}$ is transformed into a common embedding space $R^{D}$ using a learnable projection matrix $W_{i}\in R^{D\times d_{i}}$ :

$$h_{i}=W_{i}x_{i}\;\text{for each}\;i=1,2,\ldots ,M,$$

where $h_{i}\in R^{D}$ represents the projected embeddings.

Next, an attention mechanism computes the attention scores for each projected embedding $h_{i}$ . The attention score $\alpha_{i}$ for the i -th embedding is calculated as follows:

$$\alpha_{i}=\frac{\exp\left(q^{⊤}h_{i}\right)}{\sum_{j=1}^{M}\exp\left(q^{⊤}h_{j}\right)},$$

where $q\in R^{D}$ is a learnable query vector and $\alpha_{i}$ represents the normalized attention score for the i -th embedding.

The final unified embedding h is obtained by computing a weighted sum of the projected embeddings $h_{i}$ based on their attention scores:

$$h=\overset{M}{\underset{i=1}{\sum }}\alpha_{i}h_{i},$$

where $h\in R^{D}$ is the fused representation that integrates information from all modalities.

#### 4.3.2 Relation-guided dual adaptive fusion (ReDAF)

Given the sparse nature of PrimeKG++, we utilize the Relation-guided Dual Adaptive (Zhang et al., 2024) Fusion model which produces a joint embedding projected from weighted parameters collected from individual modal training data. In addition, the missing values of any element are consolidated with a random vector within the same vector space.

$$\omega_{m}\left(v,r\right)=\frac{\exp\left(V⊙\tanh\left(v_{m}\right)/\sigma \left(\zeta_{r}\right)\right)}{\sum_{n\in M\cup \left\{S\right\}}\exp\left(V⊙\tanh\left(v_{n}\right)/\sigma \left(\zeta_{r}\right)\right)},$$

where V is a learnable vector and ⊙ is the point-wise operator.

Tanh () is the tanh function. σ represents the sigmoid function to limit the relational-wise temperature in (0, 1), aiming to amplify the differences between different modal weights. With the adaptive weights, the joint embedding of an entity v is aggregated as:

$$v_{\text{joint}}=\underset{m\in M\cup \left\{S\right\}}{\sum }\omega_{m}\left(v,r\right)v_{m},$$

where $\sigma \left(x\right)=\frac{1}{1+e^{-x}}$ represents the sigmoid function, $X_{i}$ are the input features from the i -th modality, $S_{i}$ are subtype embeddings, $W_{t}$ and $W_{r}$ are transformation matrices for input features and relational context C , respectively, and $w_{i}$ are the adaptive weights for each modality. The ReLU function is fully expressed as the maximum between zero and its input, integrating the features under a non-linear transformation.

### 4.4 Graph contrastive learning

We employ GCL models to maximize the agreement between two augmented views of the same graph, facilitating the extraction of valuable insights among nodes of identical types. We specifically explore various GCL models that are suitable for Knowledge Graphs, including Deep Graph Infomax (DGI) (Veličković et al., 2018), Graph Group Discrimination (GGD) (Zheng et al., 2022), and Graph Contrastive Representation Learning (GRACE) (Zhu et al., 2020). Each of these models employs different strategies for contrastive learning, which we detail in Sections 4.4.1 - 4.4.3. Regarding augmentation techniques, while the diffusion method has demonstrated superior effectiveness (Hassani and Khasahmadi, 2020), it also demands more execution time compared to alternatives. Therefore, for the sake of efficiency, we opt to mask out nodes and remove edges randomly for fast experimentation.

#### 4.4.1 Deep graph infomax model

Deep Graph Infomax (DGI) (Veličković et al., 2018) utilizes an unsupervised learning strategy for graph data by maximizing mutual information between node representations and a global summary of the graph. The method begins with the assumption of a set of node features $X=\left\{x_{1},x_{2},\ldots ,x_{N}\right\}$ , where N is the number of nodes, and each $x_{i}\in R^{F}$ denotes the features of node i . These are complemented by an adjacency matrix $A\in R^{N\times N}$ , which encodes the relational structure between nodes.

The core of DGI is an encoder function $E:R^{N\times N}\times R^{N\times F}\to R^{N\times F_{0}}$ that transforms the node features and the adjacency matrix into high-level node embeddings $\left\{h_{1},h_{2},\ldots ,h_{N}\right\}$ . These embeddings, or patch representations, are meant to encapsulate not only the properties of individual nodes, but also their neighborhood structures.

To capture the global structure of the graph, DGI uses a readout function $R:R^{N\times F}\to R^{F}$ to aggregate these patch representations into a summary vector s=R(E(X,A)) . This vector serves as a comprehensive representation of the entire graph’s topology and feature distribution.

DGI employs a discriminator $D:s^{T}s$ , which evaluates mutual information between local patch representations and global summary by assigning probability scores. These scores indicate how well the local patches (node embeddings) and the global summary correspond to each other in terms of information content.

For training, negative samples are generated through a stochastic corruption function $C:R^{N\times N}\times R^{N\times F}\to R^{M\times M}\times R^{M\times F}$ , creating perturbed versions of the graph $\left(X_{e},A_{e}\right)=C\left(X,A\right)$ . The learning objective is to discriminate between the “true” patch-summary pairs and those generated from corrupted inputs using a noise-contrastive estimation with a binary cross-entropy loss.

This setup ensures that the encoder and discriminator learn to retain and emphasize features that are important across the graph, facilitating the discovery of intricate patterns and structural roles within the network, which can significantly enhance performance on downstream tasks like node classification.

#### 4.4.2 Graph group discrimination model

We experiment with a Group-discrimination-based method called Graph Group Discrimination (GGD) (Zheng and Li, 2022). Contrastive learning in this method is formulated to discriminate between groups of node embeddings, rather than individual pairs. This method leverages a binary cross-entropy loss to effectively distinguish between node samples from ‘positive’ (unaltered) and ‘negative’ (altered) graph structures.

Formally, in the GGD module, a graph autoencoder framework is employed to learn embeddings that are predictive of the graph structure. The nodes $v_{i}\in V$ are assigned to the embeddings $z_{i}$ using a GCN encoder E . The model then predicts the presence or absence of edges between pairs of nodes by computing logits ${\hat{y}}_{ij}=z_{i}^{T}z_{j}$ . The binary cross-entropy loss is used to train the model:

$$L\left(\theta \right)=-\underset{\left(v_{i},v_{j}\right)\in E}{\sum }\log\left(\sigma \left({\hat{y}}_{ij}\right)\right)-\underset{\left(v_{i},v_{j}\right)\notin E}{\sum }\log\left(1-\sigma \left({\hat{y}}_{ij}\right)\right),$$

where σ denotes the sigmoid function.

The primary advantage of GGD is its efficiency, especially in scenarios involving large-scale graph datasets, where it reduces computational overhead and significantly accelerates the training process. By applying this approach, our model can achieve rapid convergence and robust performance even with minimal training epochs.

#### 4.4.3 Graph contrastive representation learning model

GRACE (Graph Contrastive Representation Learning) (Zhu et al., 2020) applies stochastic augmentations to the node features and the graph structure to learn robust node embeddings. For a graph with feature matrix X and adjacency matrix A , two corrupted views $X_{1},A_{1}$ and $X_{2},A_{2}$ are generated by independently dropping features and edges. The node embeddings for these views are computed as $Z_{1}=E\left(X_{1},A_{1}\right)$ and $Z_{2}=E\left(X_{2},A_{2}\right)$ , using the same encoder E . Each embedding vector is then projected through a two-layer network with RELU activations to align the representations from different views while maintaining discriminative features. The contrastive loss, specifically the InfoNCE loss, is applied to align these representations while also distinguishing them from negatives within their minibatch:

$$L\left(\theta \right)=-\overset{n}{\underset{i=1}{\sum }}\log\frac{\exp\left(P{\left(z_{1i}\right)}^{T}P\left(z_{2i}\right)/\tau \right)}{\sum_{j=1}^{n}\exp\left(P{\left(z_{1i}\right)}^{T}P\left(z_{2j}\right)/\tau \right)},$$

where τ is a temperature scaling parameter.

### 4.5 Link prediction in KG embedding

KG Embedding involves an embedding function e:E∪R→X , which maps entities and relations in a knowledge graph to elements within an embedding space X . In addition, it includes a scoring function $f:X^{3}\to R$ that, given the embeddings of entities and relations in a triple, computes a score indicating the likelihood or validity of the triple. In our experiment, we use the Relational Graph Convolutional Network (RGCN) (Schlichtkrull et al., 2018) as the encoder to extract embeddings from graph-structured data that includes relational information. We then employ DistMult (Yang et al., 2014) as a scoring function to map entities and relations to vector scores. The essence of the link prediction task lies in classifying the existence of edges between entities, where positive edges are drawn from the dataset, and negative edges are randomly sampled. Binary Cross Entropy (BCE) loss is employed to evaluate the effectiveness of the classification as follows:

$$L_{\text{BCE}}=-\frac{1}{N}\overset{N}{\underset{i=1}{\sum }}\left[y_{i}\log\left({\hat{y}}_{i}\right)+\left(1-y_{i}\right)\log\left(1-{\hat{y}}_{i}\right)\right],$$

where N denotes the total number of training samples (triples), $y_{i}\in \left\{0,1\right\}$ is the ground-truth label indicating whether the triple $\left(h_{i},r_{i},t_{i}\right)$ exists in the knowledge graph (1 for positive edges, 0 for negative edges), and ${\hat{y}}_{i}\in \left[0,1\right]$ is the predicted probability obtained from the scoring function $f\left(e\left(h_{i}\right),e\left(r_{i}\right),e\left(t_{i}\right)\right)$ after applying a sigmoid activation.

The regularization term is added to avoid overfitting, and is given by the sum of squared norms of the latent representations and the relation embeddings:

$$L_{\text{reg}}=\lambda \left(\|X{\|}^{2}+\|Z{\|}^{2}\right),$$

where X represents the encoded entity embeddings and Z denotes the relation embeddings. The final loss function combines the binary cross-entropy loss and the weighted regularization term:

$$L=L_{\text{BCE}}+\alpha L_{\text{reg}}.$$

To facilitate effective batch-wise training, we utilize the GraphSAINT sampling method (Zeng et al., 2019), which employs the Random Walk technique to sample subgraphs while maintaining a representative distribution of existing edges within each batch for the link prediction task.

## 5 Experiments

### 5.1 Experimental setup

#### 5.1.1 Materials

In our experiments, we utilized two principal datasets: PrimeKG++ and the DrugBank drug-target interaction dataset (Knox et al., 2024). PrimeKG++ serves as our primary dataset, enriched with detailed attribute information across a variety of biological entities, making it highly suitable for comprehensive model training and evaluation. The DrugBank dataset, a curated biomedical knowledge graph, focuses specifically on drug-target protein interactions. It comprises 9,716 FDA-approved drugs and 846 protein targets, encompassing a different set of relations and nodes compared to PrimeKG++. However, the DrugBank dataset originally lacked node attributes, necessitating augmentation by incorporating detailed attribute information similar to that in PrimeKG++, thereby ensuring a comprehensive evaluation and robust performance of our model. By leveraging the enriched attribute information integrated into both datasets, we aim to thoroughly evaluate our framework’s ability to handle both broad and domain-specific biomedical knowledge graphs, enabling a rigorous assessment of its performance and generalizability.

#### 5.1.2 Comparative analysis of embedding techniques on PrimeKG++

With the introduction of PrimeKG++, our augmented dataset, we conducted a comprehensive evaluation of our approach by exploring a variety of widely-used configurations. We experimented with three well-established GCL models: Graph Group Discrimination (GGD), Graph Contrastive Representation Learning (GRACE), and Deep Graph Infomax (DGI). Additionally, we examined different attribute fusion methods, including Attention Fusion and Relation-guided Dual Adaptive Fusion (ReDAF), which weigh each modality differently before fusion. As a baseline, we also included a simple fusion approach (“None”) where embeddings from various modalities were combined using a mean operation without explicit weighting. To provide additional context, we compared these configurations against models trained with Random Initialization and direct Language Model (LM)-derived embeddings. Rather than focusing on identifying a single optimal configuration, our objective was to demonstrate the versatility and robustness of the proposed approach across widely-used methods. We experimented with different configurations to show how our framework can be applied in diverse settings. Although the choice of components may depend on the specific characteristics of the dataset, our intention was to highlight the adaptability of our framework, ensuring that it performs effectively in multiple configurations.

#### 5.1.3 Evaluating generalizability on the DrugBank dataset

To assess the robustness and generalizability of our framework, we conducted extensive experiments on the DrugBank drug-target interaction (DTI) dataset. Our approach utilizes GCL models pre-trained in PrimeKG++ to generate initial embeddings, providing a rich semantic and relational foundation. These embeddings are then fine-tuned using Knowledge Graph Embedding (KGE) models, specifically optimized for each configuration, on the training set of the DrugBank DTI dataset. This two-step process ensures that pre-trained embeddings effectively capture meaningful information from PrimeKG++ while adapting to the unique relational and attribute structures of DrugBank. By evaluating performance across various configurations, we demonstrate our framework’s ability to generalize to novel entities and its effectiveness in handling datasets with diverse relational and attribute characteristics.

#### 5.1.4 Implementation details

For our experiments, we randomly split the edges of PrimeKG++ and the DrugBank drug-target interaction dataset into three subsets: training, validation, and testing, with a corresponding ratio of 60:20:20. This ensures a balanced and comprehensive evaluation of our model across both datasets. The PrimeKG++ dataset provides a richly augmented set of node attributes, while the DrugBank dataset serves as a complementary benchmark for evaluating the model’s generalizability to unseen nodes and distinct relational structures. In both cases, consistent hyperparameters and settings were applied to ensure a fair and rigorous evaluation process.

To further challenge the model and assess its robustness, we adjusted the negative sampling ratio in our experiments. Although the standard ratio is 1:1 (one negative sample for each positive sample), we increase this ratio to 1:3 and 1:5 in certain configurations. These higher ratios create significantly more difficult tasks by introducing a larger set of negative edges, testing the model’s ability to distinguish true interactions from a broader range of false ones. This adjustment enables a deeper evaluation of the model’s performance in scenarios closer to real-world conditions, where true interactions are relatively sparse.

The reported results are based on models with the lowest validation loss observed during training, evaluated over 100 epochs. The statistics of the dataset splits are summarized in Table 3. Our model implementations are built using PyTorch and trained on a single NVIDIA A100 GPU for 3 h for training. Detailed settings for all hyperparameters and summary of our models are provided in Tables 4, 5. This setup ensures a rigorous and reproducible evaluation framework for assessing the performance and generalizability of our proposed methods.

**TABLE 3:** Statistics of triple splits for PrimeKG++ and DPI benchmark.

Dataset	Total	Training	Validation	Testing
PrimeKG++	3,527,861	2,116,717	705,572	705,572
DTI benchmark	42,012	25,208	8,402	8,402

**TABLE 4:** List of hyperparameters.

Hyperparameter	Description	Value
Learning rate	Step size for updates	0.001
Batch size	Samples per update	128
Epochs	Passes through dataset	100
Embed dim	Initial embedding size	768
Hidden dim	Hidden layer size	128
Hidden layers	Number of hidden layers	2
Dropout rate	Fraction of units to drop	0.2
Embedding dimension	Size of embeddings	128
Regularization weight (λ)	Weight for regularization	0.01
Random walk length	Length of random walks	10
Random walk step	Number of random walks step	1,000
Optimizer	Optimization algorithm	Adam
Learning rate schedule	Schedule for learning rate	Cosine annealing
Warm-up steps	Steps for learning rate warm-up	200
Activation function	Activation function	ReLU
Gradient clipping	Max gradient norm	1.0
Early stopping patience	Epochs with no improvement	3

**TABLE 5:** Model summary.

Category	Model	No. Parameters
GCL	GCN encoder	164K
Modality fusion	Attention	1.8 M
	ReDAF	1.2 M
KGE	RGCN	590K

#### 5.1.5 Evaluation metrics

To assess the effectiveness of our model in the link prediction task, we employ two widely recognized metrics: Average Precision (AP) and F1-score. AP provides a comprehensive measure of precision across recall levels, making it suitable for imbalanced datasets and varying negative sampling ratios. F1-score, the harmonic mean of precision and recall, captures the balance between false positives and false negatives, offering an interpretable measure of classification performance. These metrics ensure a robust assessment of the model’s effectiveness in link prediction tasks across various experimental settings.

### 5.2 Results and discussion

#### 5.2.1 PrimeKG++

Table 6 shows that embeddings derived from pre-trained language models (LMs) consistently outperform those from random initialization, highlighting the value of external knowledge in link prediction. Building on this, our framework which integrates LM-derived embeddings with relational insights through GCL achieves the best overall performance across all settings. Notably, GRACE with ReDAF delivers the strongest results, reaching an AP of 0.996 and F1 of 0.983 under a 1:1 ratio, and maintaining robust performance under more challenging negative sampling ratios (AP/F1 of 0.988/0.947 at 1:3 and 0.980/0.916 at 1:5). While LM-only embeddings provide a strong initialization (AP 0.993, F1 0.975 at 1:1), their performance drops more sharply as sampling becomes harder.

**TABLE 6:** Link prediction performance on the PrimeKG++ dataset with varying negative sampling ratios.

Initial embedding	Attribute fusion	GCL models	1:1		1:3		1:5	
			AP	F1	AP	F1	AP	F1
Random initialization	—	—	0.980	0.960	0.945	0.893	0.909	0.829
Direct LM-derived	None	—	0.993	0.975	0.982	0.934	0.972	0.902
Our approaches	None	GGD	0.993	0.978	0.979	0.933	0.966	0.895
	Attention		0.994	0.979	0.982	0.937	0.970	0.901
	ReDAF		0.993	0.978	0.981	0.934	0.968	0.896
	None	GRACE	**0.996**	**0.983**	0.987	**0.947**	0.979	0.916
	Attention		**0.996**	**0.983**	0.982	0.937	**0.980**	**0.917**
	ReDAF		**0.996**	**0.983**	**0.988**	**0.947**	0.980	0.916
	None	DGI	0.993	0.979	0.980	0.936	0.968	0.899
	Attention		0.994	0.979	0.982	0.936	0.970	0.898
	ReDAF		0.993	0.977	0.979	0.931	0.965	0.891

Bold values indicate the best result for each metric.

#### 5.2.2 DrugBank DTI

As shown in Table 7, we evaluate our framework on the DrugBank drug-target interaction (DTI) dataset to test its generalization to unseen nodes and distinct relational structures. Models trained from random initialization perform the weakest, with performance dropping sharply as task difficulty increases: from an AP of 0.834 and F1 of 0.749 at a 1:1 negative sampling ratio to an AP of 0.579 and F1 of 0.591 at 1:5. This steep decline underscores the limitations of training from scratch without prior semantic knowledge. By contrast, embeddings derived from pre-trained language models (LMs) deliver a substantial boost, reaching an AP of 0.994 and F1 of 0.957 at 1:1, and still achieving AP 0.982 and F1 0.822 under the most challenging 1:5 setting. These results highlight the importance of external biomedical knowledge and confirm that LM-based initialization provides a strong foundation for link prediction.

**TABLE 7:** Link prediction performance on the DrugBank DTI dataset with varying negative sampling ratios.

Initial embedding	Attribute fusion	GCL models	1:1		1:3		1:5	
			AP	F1	AP	F1	AP	F1
Random initialization	—	—	0.834	0.749	0.661	0.513	0.579	0.591
Direct LM-derived	None	—	0.994	0.957	**0.988**	0.884	**0.982**	0.822
Our approaches	None	GGD	0.985	0.948	0.963	0.862	0.936	0.793
	Attention		0.9862	0.951	0.964	0.870	0.940	0.803
	ReDAF		0.9865	0.954	0.965	0.877	0.941	0.813
	None	GRACE	**0.994**	**0.972**	0.985	**0.928**	0.976	**0.887**
	Attention		**0.994**	**0.972**	0.986	0.927	0.976	**0.887**
	ReDAF		**0.994**	0.969	0.986	0.918	0.977	0.871
	None	DGI	0.986	0.948	0.964	0.863	0.940	0.793
	Attention		0.986	0.95	0.966	0.870	0.943	0.803
	ReDAF		0.983	0.946	0.957	0.858	0.928	0.785

Bold values indicate the best result for each metric.

Building on this foundation, our proposed framework, which integrates LM-derived embeddings with GCL, achieves the best overall results across all configurations. GRACE variants yield the highest scores, with AP/F1 of 0.994/0.972 at 1:1 and maintaining strong performance at 1:5 (0.976/0.887), demonstrating robustness under increasing negative sampling ratios.

#### 5.2.3 Discussion

Tables 6, 7 show that LM-derived embeddings consistently outperform random initialization, underscoring the value of leveraging pretrained biomedical knowledge from PrimeKG. Adding GCL modules further refines these embeddings by enhancing relational consistency and structural robustness. However, the gains over simple mean pooling are relatively modest (typically within 0.1%–0.2%) when compared to more expressive fusion mechanisms such as Attention and ReDAF.

To better assess their impact, we conducted an additional experiment (Section 5.4.2) where node embeddings were derived from the PrimeKG++ pretrained setting, with initialization strategies including random, LM, and GCL combined with different fusion methods. These embeddings were then frozen and evaluated using ML models only. As shown in Table 10, Attention and ReDAF yield 1%–3% improvements over mean pooling across multiple GCL backbones, highlighting the effectiveness of GCL in producing more informative embeddings.

### 5.3 Latent space visualization of embeddings

To assess embedding quality, we performed a latent space visualization using the PrimeKG++ dataset, which was used during GCL model pre-training. Visualizing the entire dataset is challenging due to the complexity of link prediction tasks and the difficulty in interpreting dense patterns. Therefore, we concentrated on the protein with the highest number of interactions, allowing us to present a focused and meaningful visualization that reflects the relational and semantic structure relevant to the link prediction objective.

Using t-SNE, we projected the high-dimensional drug embeddings into a 2D space. The embeddings were categorized into two groups: drugs that interact with the selected protein and drugs that do not interact with the protein. To evaluate the effectiveness of our approach, we compared embeddings generated through two configurations: Language Model (LM)-based embeddings and enhanced embeddings through our proposed method. For our approach, we employed GRACE + ReDAF, which is our most stable configuration, effectively combining LM and Graph Contrastive Learning (GCL) to incorporate relational information.

**FIGURE 3:**
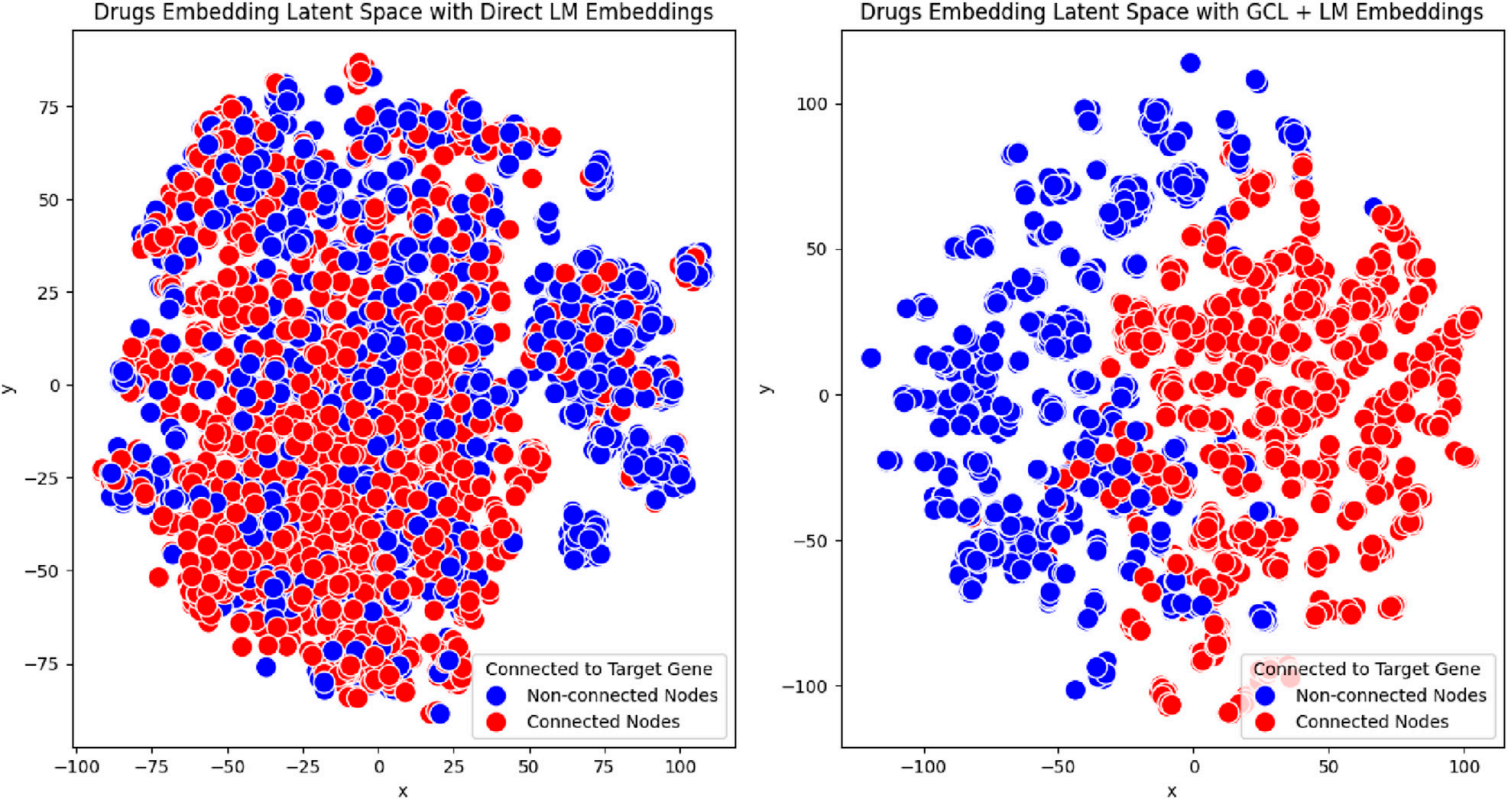
t-SNE visualization of drug embeddings for a single protein with the highest number of interactions in the PrimeKG++ dataset. The left panel displays embeddings derived solely from the Language Model (LM), while the right panel shows embeddings generated using our proposed approach (GRACE + ReDAF). Drugs interacting with the selected protein are labeled in red, and non-interacting drugs are labeled in blue. This comparison illustrates the structural differences in the latent space resulting from the two embedding methods.

The visualization results in Figure 3 reveal notable differences between the two configurations. Embeddings generated solely with Language Models (LM) showed less distinct clustering, with considerable overlap between the two groups. This overlap suggests a limited ability to distinguish drugs that interact with the selected protein from those that do not.

In contrast, embeddings produced using our proposed method, which integrates LM with Graph Contrastive Learning (GCL), exhibited tighter clustering and more pronounced separation. This demonstrates the method’s superior ability to capture shared properties among drugs interacting with the same protein.

These results underscore the robustness of our framework in generating high-quality, interpretable embeddings that accurately represent the underlying biological relationships, even when applied to unseen datasets such as DrugBank.

### 5.4 Additional results

In this section, we present additional experimental results to assess the effectiveness of our proposed approach and its components. First, we examine the impact of embedding size, showing that larger embeddings lead to improved performance. Next, we evaluate precision across different relation types, demonstrating that our model performs well in distinguishing between true and false relationships. Finally, we assess embedding quality in downstream tasks, where our approach, combining intra- and inter-learning, yields better embeddings that contribute to stronger task performance. These findings offer valuable insights to support future research in this area.

#### 5.4.1 Impact of embedding size on model performance

The size of the embedding plays a critical role in model performance, as it determines the capacity to capture complex features of the data. To identify the optimal configuration and understand the trade-off between embedding size and performance, we systematically evaluate the impact of various embedding sizes using the Grace-Attention model. This experimentation provides insights into how embedding dimensionality influences the model’s capacity and effectiveness.

The results, summarized in Table 8, indicate that as the embedding size increases, both F1-score and AP improve, indicating that larger embeddings capture more information, leading to better performance. However, the performance improvement between 128 and 256 is marginal, suggesting diminishing returns for increasing embedding size beyond a certain threshold.

**TABLE 8:** Impact of embedding size on link prediction performance.

Embedding size	No. parameters	AP	F1
64	258K	0.988	0.97
128	738K	0.994	0.98
256	2 M	0.996	0.983

#### 5.4.2 Performance per relation type

To understand how our approach generalizes across different biomedical relationships, we evaluate the performance of the model for each type of relation within PrimeKG++ using the Grace-Attention model. The primary evaluation metric used are Average Precision and F1-score, as it provides a stable and clear measure of performance, particularly given the variability in the number of negative edges due to random negative sampling. This allows us to assess how well our model differentiates true relationships (true positives) from incorrect predictions (false positives) across diverse types of relations. To further examine robustness and generalizability, we train and test the model using a 1:10 negative sampling ratio.

The results, summarized in Table 9, present the precision values for each relation type in PrimeKG++. Our findings indicate that the Grace-Attention model maintains high precision across all relation types, regardless of the size of the relation set. In particular, the high precision in predicting drug-protein interactions suggests that the model is highly effective in identifying accurate associations between drugs and proteins, which is critical for drug repurposing. Such precise predictions can help to discover new therapeutic uses for existing drugs and identify potential drug interactions, ultimately supporting more targeted and efficient drug development efforts.

**TABLE 9:** Precision per relation type in PrimeKG++ using the Grace-Attention model.

Relation type	Precision	Number of positive edges
Contraindication	0.991	61,350
Disease-disease	0.954	64,388
Disease-protein	0.867	160,822
Drug-drug	0.974	2,672,628
Drug-protein	0.995	51,306
Indication	0.989	18,776
Off-label use	0.994	4,429,078
Protein-protein	0.911	642,150

#### 5.4.3 Evaluating embedding quality for downstream tasks

To further assess the effectiveness of node embeddings in downstream tasks, specifically DrugBank DTI, we initialize embeddings with output from a Knowledge Graph Embedding (KGE) model and train a machine learning model using XGBoost. The XGBoost model is configured with 500 estimators and a learning rate of 0.01. To ensure robust evaluation, we use a stratified 5-fold cross-validation approach, where metrics are reported as the mean performance across all folds.

Although link prediction has previously been performed in our study to evaluate embedding quality, it focuses primarily on reconstructing known relationships within the graph. In contrast, training a machine learning model for a downstream task allows us to assess whether embeddings effectively capture task-specific patterns and generalize beyond the original graph structure, providing a more comprehensive evaluation of embedding quality.

The results in Table 10 highlight that our framework, which integrates self-supervised intra-learning through Graph Contrastive Learning (GCL) and inter-learning via the link prediction task, significantly outperforms both random initialization and Direct-LM embeddings. GCL consistently achieves higher performance, showcasing its effectiveness in capturing richer and more comprehensive embeddings.

**TABLE 10:** Comparison of embedding methods for ML downstream task.

Embedding	GCL models	Fusion	AP	F1
Random from scratch			0.233	0
Random	—	—	0.508	0.509
LM	—	—	0.555	0.56
Our approach	GGD	None	0.612	0.624
		Attention	**0.646**	**0.656**
		ReDAF	0.634	0.651
	GRACE	None	0.621	0.601
		Attention	0.636	0.611
		ReDAF	0.612	0.608
	DGI	None	0.633	0.625
		Attention	0.64	0.645
		ReDAF	0.639	0.642

Bold values indicate the best result for each metric.

Compared to training from scratch, where each node is initialized randomly, our approach delivers superior results across multiple configurations, emphasizing the critical role of embedding quality in downstream tasks. For future development, this framework can serve as a baseline for initializing embeddings in machine learning models, significantly reducing resource usage while maintaining strong performance.

## 6 Conclusion

In this article, we present a novel pre-training node representation model designed to enhance link prediction performance in Biomedical Knowledge Graphs (BKG). Our approach combines semantic information from node attributes with relational data from PrimeKG++, producing robust and meaningful node embeddings. By incorporating multimodal data, such as biological sequences and textual descriptions, we enrich the contextual understanding of relationships within the graph. Furthermore, we leveraged Graph Contrastive Learning (GCL) in combination with Language Models (LMs) to optimize intra-node relationships, resulting in more generalizable embeddings capable of handling unseen nodes.

To address the issue of sparse node attributes in existing BKGs, we introduced PrimeKG++, an enriched biomedical knowledge graph that integrates biological sequences and detailed textual descriptions across various entity types. This enhancement not only resolves the limitations of PrimeKG, but also serves as a valuable resource for advancing research in the field. Furthermore, experiments conducted in PrimeKG++ demonstrate that our pre-trained node representations significantly outperform baselines, including random initialization and direct LM-derived embeddings, highlighting the advantage of combining semantic and relational information for improved link prediction.

To further validate our framework, we evaluated it on the DrugBank drug-target interaction (DTI) dataset, showcasing its strong generalization capabilities. Despite the distinct set of relations and unseen nodes in the dataset, our approach consistently outperformed baseline methods, demonstrating robust performance even under more challenging scenarios. Importantly, while this work focused on drug-protein interactions as the primary use case, the flexibility of our framework allows it to be easily extended to other relationship types, such as drug-disease or protein-disease interactions, further broadening its applicability.

This work makes substantial contributions to the field, particularly through the development of PrimeKG++, a comprehensive multimodal knowledge graph that integrates detailed biological sequences and textual descriptions, addressing key limitations of prior datasets. Our pre-trained node attributes encoder, which will be made publicly available, provides a valuable tool for researchers, enabling them to directly leverage high-quality embeddings for their own work. The versatility and adaptability of our framework make it well-suited for application across diverse multimodal knowledge graphs, underscoring its broader impact in advancing biomedical knowledge representation and discovery.

## Data Availability

The datasets presented in this study can be found in online repositories. The names of the repository/repositories and accession number(s) can be found in the article/supplementary material.

## References

[B1] Brandes N. Ofer D. Peleg Y. Rappoport N. Linial M. (2022). ProteinBERT: a universal deep-learning model of protein sequence and function. Bioinformatics 38, 2102–2110. 10.1093/bioinformatics/btac020 35020807 PMC9386727

[B2] Chandak P. Huang K. Zitnik M. (2023). Building a knowledge graph to enable precision medicine. Sci. Data 10, 67. 10.1038/s41597-023-01960-3 36732524 PMC9893183

[B3] Chen H. (2023). Large knowledge model: perspectives and challenges. arXiv preprint arXiv:2312.02706.

[B4] Chilingaryan G. Tamoyan H. Tevosyan A. Babayan N. Hambardzumyan K. Navoyan Z. (2024). Bartsmiles: generative masked language models for molecular representations. J. Chem. Inf. Model. 64 (15), 5832–5843. 39054761 10.1021/acs.jcim.4c00512

[B5] Dalla-Torre H. Gonzalez L. Mendoza-Revilla J. Carranza N. L. Grzywaczewski A. H. Oteri F. (2023). The nucleotide transformer: building and evaluating robust foundation models for human genomics. bioRxiv. 10.1101/2023.01.11.523679 PMC11810778 39609566

[B6] Daza D. Alivanistos D. Mitra P. Pijnenburg T. Cochez M. Groth P. (2023). Bioblp: a modular framework for learning on multimodal biomedical knowledge graphs. J. Biomed. Semant. 14, 20. 10.1186/s13326-023-00301-y 38066573 PMC10709903

[B7] Devlin J. Chang M.-W. Lee K. Toutanova K. (2019). BERT: pre-training of deep bidirectional transformers for language understanding. 4171, 4186. 10.18653/v1/N19-1423

[B8] Fu H. Huang F. Liu X. Qiu Y. Zhang W. (2021). MVGCN: data integration through multi-view graph convolutional network for predicting links in biomedical bipartite networks. Bioinformatics 38, 426–434. 10.1093/bioinformatics/btab651 34499148

[B9] Hansel K. Dudgeon S. N. Cheung K.-H. Durant T. J. Schulz W. L. (2023). From data to wisdom: biomedical knowledge graphs for real-world data insights. J. Med. Syst. 47, 65. 10.1007/s10916-023-01951-2 37195430 PMC10191934

[B10] Hassani K. Khasahmadi A. H. (2020). Contrastive multi-view representation learning on graphs. International conference on machine learning. PMLR. 4116–4126.

[B11] He Q. Wang Y. Wang W. (2024). Can language models act as knowledge bases at scale? arXiv preprint arXiv:2402.14273.

[B12] Himmelstein D. S. Lizee A. Hessler C. Brueggeman L. Chen S. L. Hadley D. (2017). Systematic integration of biomedical knowledge prioritizes drugs for repurposing. Elife 6, e26726. 10.7554/eLife.26726 28936969 PMC5640425

[B13] Ji Y. Zhou Z. Liu H. Davuluri R. V. (2021). DNABERT: pre-trained bidirectional encoder Representations from transformers model for DNA-language in genome. Bioinformatics 37, 2112–2120. 10.1093/bioinformatics/btab083 33538820 PMC11025658

[B14] Jiang Z. Sun Z. Shi W. Rodriguez P. Zhou C. Neubig G. (2024). Instruction-tuned language models are better knowledge learners. 5421, 5434. 10.18653/v1/2024.acl-long.296

[B15] Knox C. Wilson M. Klinger C. M. Franklin M. Oler E. Wilson A. (2024). Drugbank 6.0: the drugbank knowledgebase for 2024. Nucleic Acids Research 52, D1265–D1275. 10.1093/nar/gkad976 37953279 PMC10767804

[B16] Lam H. T. Sbodio M. L. Gallindo M. M. Zayats M. Fernandez-Diaz R. Valls V. (2023). Otter-knowledge: benchmarks of multimodal knowledge graph representation learning from different sources for drug discovery. arXiv Preprint arXiv:2306.12802.

[B17] Lee J. Yoon W. Kim S. Kim D. Kim S. So C. H. (2020). Biobert: a pre-trained biomedical language representation model for biomedical text mining. Bioinformatics 36, 1234–1240. 10.1093/bioinformatics/btz682 31501885 PMC7703786

[B18] Lewis P. Ott M. Du J. Stoyanov V. (2020). “Pretrained language models for biomedical and clinical tasks: understanding and extending the state-of-the-art,” in Proceedings of the 3rd clinical natural language processing workshop. Editors Rumshisky A. Roberts K. Bethard S. Naumann T. (Stroudsburg, PA: Association for Computational Linguistics), 146–157. 10.18653/v1/2020.clinicalnlp-1.17

[B19] Lin Z. Zhang Z. Wang M. Shi Y. Wu X. Zheng Y. (2022). Multi-modal contrastive representation learning for entity alignment. arXiv preprint arXiv:2209.00891.

[B20] Lin Z. Akin H. Rao R. Hie B. Zhu Z. Lu W. (2023). Evolutionary-scale prediction of atomic-level protein structure with a language model. Science 379, 1123–1130. 10.1126/science.ade2574 36927031

[B21] Maglott D. Ostell J. Pruitt K. D. Tatusova T. (2010). Entrez gene: gene-centered information at ncbi. Nucleic Acids Research 39, D52–D57. 10.1093/nar/gkq1237 21115458 PMC3013746

[B22] Menon A. K. Elkan C. (2011). “Link prediction *via* matrix factorization,” in Machine learning and knowledge discovery in databases. Editors Gunopulos D. Hofmann T. Malerba D. Vazirgiannis M. (Berlin, Heidelberg: Springer Berlin Heidelberg), 437–452.

[B23] Ngo K. N. Hy T. S. Kondor R. (2022). “Predicting drug-drug interactions using deep generative models on graphs,” in NeurIPS 2022 AI for science: progress and promises.

[B24] Nicholson D. N. Greene C. S. (2020). Constructing knowledge graphs and their biomedical applications. Comput. Struct. Biotechnol. J. 18, 1414–1428. 10.1016/j.csbj.2020.05.017 32637040 PMC7327409

[B25] Peng Z. Huang W. Luo M. Zheng Q. Rong Y. Xu T. (2020). Graph representation learning *via* graphical mutual information maximization. 259, 270. 10.1145/3366423.3380112

[B26] Petroni F. Rocktäschel T. Lewis P. Bakhtin A. Wu Y. Miller A. H. (2019). Language models as knowledge bases? arXiv Preprint arXiv:1909, 01066.

[B27] Ross J. Belgodere B. Chenthamarakshan V. Padhi I. Mroueh Y. Das P. (2022). Large-scale chemical language representations capture molecular structure and properties. Nat. Mach. Intell. 4, 1256–1264. 10.1038/s42256-022-00580-7

[B28] Schlichtkrull M. Kipf T. N. Bloem P. van den Berg R. Titov I. Welling M. (2018). Modeling relational data with graph convolutional networks. European semantic web conference. 593–607.

[B29] Sun Z. Deng Z.-H. Nie J.-Y. Tang J. (2019). Rotate: knowledge graph embedding by relational rotation in complex space. arXiv Preprint arXiv:1902, 10197.

[B30] Trouillon T. Welbl J. Riedel S. Gaussier É. Bouchard G. (2016). “Complex embeddings for simple link prediction,” in International conference on machine learning (PMLR), 2071–2080.

[B31] Vaswani A. Shazeer N. Parmar N. Uszkoreit J. Jones L. Gomez A. N. (2017). Attention is all you need. Adv. Neural. Inf. Process. Syst. 30.

[B32] Veličković P. Fedus W. Hamilton W. L. Lió P. Bengio Y. Hjelm R. D. (2018). Deep graph infomax. arXiv preprint arXiv:1809.10341.

[B33] Walsh B. Mohamed S. K. Nováček V. (2020). “Biokg: a knowledge graph for relational learning on biological data,” in Proceedings of the 29th ACM international conference on information and knowledge management, 3173–3180.

[B34] Wang M. Wang H. Liu X. Ma X. Wang B. (2021). Drug-drug interaction predictions *via* knowledge graph and text embedding: instrument validation study. JMIR Med. Inf. 9, e28277. 10.2196/28277 34185011 PMC8277366

[B35] Wang B. Xie Q. Pei J. Chen Z. Tiwari P. Li Z. (2023). Pre-trained language models in biomedical domain: a systematic survey. ACM Comput. Surv. 56, 1–52. 10.1145/3611651

[B36] Yang B. Yih W.-t. He X. Gao J. Deng L. (2014). Embedding entities and relations for learning and inference in knowledge bases. arXiv preprint arXiv:1412.6575.

[B37] Yang Y. Huang C. Xia L. Li C. (2022). Knowledge graph contrastive learning for recommendation. 1434, 1443. 10.1145/3477495.3532009

[B38] Zeng H. Zhou H. Srivastava A. Kannan R. Prasanna V. (2019). Graphsaint: graph sampling based inductive learning method. arXiv preprint arXiv:1907.04931.

[B39] Zhang L. Li R. (2022). KE-GCL: knowledge enhanced graph contrastive learning for commonsense question answering. Findings-Emnlp, 6. 10.18653/v1/2022

[B40] Zhang Y. Chen Z. Guo L. Xu Y. Hu B. Liu Z. (2024). Native: multi-modal knowledge graph completion in the wild. Authorea Prepr., 91–101. 10.1145/3626772.3657800

[B41] Zhao J. Zhang Z. Gao L. Zhang Q. Gui T. Huang X. (2024). Llama beyond english: an empirical study on language capability transfer. arXiv preprint arXiv:2401.01055.

[B42] Zheng Y. Pan S. Lee V. C. Zheng Y. Yu P. S. (2022). Rethinking and scaling up graph contrastive learning: an extremely efficient approach with group discrimination. Advances in Neural Information Processing Systems. 35, 10809–10820.

[B43] Zhu Y. Xu Y. Yu F. Liu Q. Wu S. Wang L. (2020). Deep graph contrastive representation learning. arXiv preprint arXiv:2006.04131.

[B44] Zitnik M. Agrawal M. Leskovec J. (2018). Modeling polypharmacy side effects with graph convolutional networks. Bioinformatics 34, i457–i466. 10.1093/bioinformatics/bty294 29949996 PMC6022705

